# Compliance with the ‘Japanese food guide spinning top’ for pregnancy and lactation and weight gain during pregnancy

**DOI:** 10.1017/jns.2026.10134

**Published:** 2026-08-03

**Authors:** Reina Doi, Minatsu Kobayashi, Kohei Ogawa, Seung Chik Jwa, Takeo Fujiwara, Hisako Tanaka, Keisuke Yoshii, Naho Morisaki

**Affiliations:** 1 Department of Human Life Sciences, Graduate School of Human Culture, Otsuma Women’s University, Japan; 2 Department of Food Science, Faculty of Home Economics, Otsuma Women’s University, Japan; 3 Center for Maternal-Fetal, Neonatal and Reproductive Medicine, National Center for Child Health and Development, Japan; 4 Department of Obstetrics and Gynecology, Jichi Medical University, Japan; 5 Department of Public Health, Institute of Science Tokyo, Japan; 6 Department of Social Medicine, National Center for Child Health and Development, Japan; 7 Division of Endocrinology and Metabolism, National Center for Child Health and Development, Japan

**Keywords:** Dietary habits, gestational weight gain, Japanese women, lactation, pregnancy

## Abstract

In Japan, a version of the Japanese Food Guide, specifically developed for pregnancy and lactation (JFGST-P/L), promotes healthy dietary habits. In the mid- to-late stages of pregnancy, it is especially important to consume the recommended additional calories and nutrients. Adherence to these guidelines also plays a key role in achieving appropriate gestational weight gain (GWG). This study investigated the relationship between adherence to these guidelines and GWG among women in the mid- to late stages of pregnancy. Data were obtained from the Maternal and Child Cohort Study, conducted at the National Center for Child Health and Development (Setagaya-ku, Tokyo, Japan). The degree of adherence to the JFGST-P/L was evaluated using scores calculated from responses to a semi-quantitative food frequency questionnaire. The participants were classified into quartiles (Q1–Q4) based on their adherence scores. GWG was classified as inadequate, adequate, or excessive according to the Ministry of Health, Labour and Welfare guidelines. The degree of JFGST-P/L adherence was divided into quartiles, and a multinomial logistic regression analysis was performed. Furthermore, the Q1 group was compared with the Q2–Q4 groups to examine the association with the risk of insufficient GWG. In the quartile analysis, no clear dose–response relationship was observed between JFGST-P/L adherence and GWG. Conversely, among Q1, an increased risk of insufficient GWG was noted compared to the Q2–Q4 group (Unadjusted OR 1.389, 95%CI 1.008–1.913). In this study, the lowest degree of adherence to the JFGST-P/L among pregnant women was associated with inadequate GWG.

## Introduction

A high prevalence of underweight status, along with insufficient energy intake and poor dietary balance, has been reported among young Japanese women.^(1–3)^ The 2023 National Health and Nutrition Survey states that approximately 20% of women in their twenties have a BMI below 18.5, and adherence to balanced meals combining staple foods, main dishes, and side dishes remains low.^(2)^ Such dietary habits tend to persist during pregnancy and may influence gestational weight gain (GWG).

The GWG is intricately associated with birth outcomes. Excessive GWG increases the risk of macrosomia and caesarean delivery, whereas inadequate GWG is associated with low birth weight (LBW) and fetal growth restriction.^(4–7)^ Notably, undernutrition *in utero* increases the risk of ischaemic heart disease and diabetes later in life.^(8)^ Japan has a higher prevalence of LBW than other countries,^(9)^ and international comparisons indicate that Japanese women tend to have particularly low GWG.^(10,11)^ Therefore, ensuring adequate GWG is an important public health concern for Japanese pregnant women.

Japan developed a Pregnancy and Lactation version of the Japanese Food Guide Spinning Top (JFGST-P/L)^(12,13)^ to support dietary improvement during pregnancy. The JFGST-P/L provides practical guidance on food groups and their recommended amounts, enabling pregnant women to better understand their overall dietary intake. Nutritional counseling based on the JFGST-P/L has been shown to increase the intake of staple foods and vegetables.^(14)^

Several studies have examined the relationship between dietary quality and GWG. Higher diet quality, as assessed by the Dietary Approaches to Stop Hypertension (DASH) diet or the Healthy Eating Index, has been associated with a lower risk of inadequate GWG,^(15)^ whereas an Alternative Mediterranean Diet has been linked to reduced excessive GWG.^(16)^ A review by the U.S. Dietary Guidelines Advisory Committeesimilarly concluded that dietary patterns rich in vegetables, fruits, legumes, nuts, and fish are associated with a lower risk of excessive GWG.^(17)^ In Japan, some studies have reported that although underweight women have a higher diet quality, they still show inadequate GWG,^(18)^ highlighting the need for dietary assessments that account for both energy intake and nutrient density.

In our previous study, we demonstrated that pregnant women with higher adherence to the JFGST-P/L had greater energy intake and higher consumption of folate, calcium, and dietary fibre, which is often insufficient during pregnancy.^(19)^ Thus, the JFGST-P/L serves as a comprehensive indicator of dietary quality during pregnancy.

Furthermore, achieving appropriate GWG during pregnancy requires not only focusing on specific foods or nutrients but also maintaining a nutritionally balanced diet by regularly combining staple foods, main dishes, and side dishes. Studies have reported that inadequate dietary balance, such as insufficient intake of main dishes from early pregnancy, may affect the GWG in pregnant women whose weight gain during pregnancy is below the recommended range.^(3)^ That is, adherence to the JFGST-P/L reflects the appropriate intake of staple foods, main dishes, side dishes, etc., and is considered to influence weight gain during pregnancy. Inadequate GWG throughout pregnancy has been reported to be associated with an increased risk of LBW.^(20)^ Particularly, the mid-to-late pregnancy period is a critical time when maternal energy and nutrient requirements increase owing to fetal growth. The association between adherence to the JFGST-P/L during mid-to-late pregnancy, when nutrient intake increases, and total GWG has not been sufficiently investigated. Therefore, this study examined the association between the degree of adherence to the JFGST-P/L assessed in mid-to-late pregnancy and total GWG throughout pregnancy. Furthermore, total GWG was classified as inadequate, adequate, or excessive. Using appropriate GWG as the reference category, its association with the degree of adherence to the JFGST-P/L was evaluated using multinomial logistic regression analysis.

## Materials and methods

### Survey period and subjects

This study used data from the Mother and Child Cohort Study at the National Center for Child Health and Development, a prospective cohort study in which pregnant women scheduled for delivery at our institution were recruited between August 2010 and March 2013. Pregnancy and childbirth data were obtained from maternity check-ups and medical records at the time of delivery. A self-administered questionnaire, which covered demographic, socioeconomic, and lifestyle factors, and a semi-quantitative food frequency questionnaire (sFFQ) were administered twice: before 26 weeks of gestation (corresponding to early and mid-pregnancy) and from 26 weeks to delivery (corresponding to mid-to-late pregnancy).

A semi-quantitative FFQ was administered twice during pregnancy (before 26 weeks of gestation and at or after 26 weeks), and habitual dietary intake over the preceding 2 months was assessed on each occasion. Although some overlap in follow-up periods may have occurred, only the FFQ administered at or after 26 weeks of gestation was used in this analysis because it was considered more representative of dietary intake during the period most relevant to the subsequent GWG. Of the 2,432 women who consented to participate during pregnancy, 1,524 completed the sFFQ at or after 26 weeks of gestation, yielding a response rate of 62.7%. The exclusion criteria were as follows: unknown sFFQ response date (*n* = 232), incomplete sFFQ responses (*n* = 10), and missing pre-pregnancy weight (*n* = 13). In this study, participants whose reported total energy intake was in the highest or lowest 1% of the distribution (i.e., less than approximately 500 kcal/day or >3,700 kcal/day, respectively) were excluded to minimize the influence of implausible extreme values (*n* = 22). These cutoff values were comparable to those used in previous analyses of the Japan Environment and Children’s Study, which excluded participants with implausibly low or high energy intake (<500 kcal/day or >4,000 kcal/day).^(21,22)^ The JFGST-P/L systematically classifies energy and nutrient requirements into three distinct stages based on fetal growth and physiological changes in the mother. Pregnancy stages were defined as follows: early pregnancy (up to 13 weeks + 6 days), mid-pregnancy (14–27 weeks + 6 days), and late pregnancy (28 weeks onward).

After applying the exclusion criteria, 1,247 participants were included in the final analysis. This study was approved by the Ethics Committee of the National Center for Child Health and Development (Approval No. 2010-417 and 2023-268).

### Baseline survey

Maternal age (years), height (cm), pre-pregnancy weight (kg), parity, and fetal sex were extracted from prenatal care and delivery records. At enrollment and again between 26 and 40 weeks of gestation, participants completed self-administered questionnaires assessing household income (<4 million yen, 4–6 million yen, 6–8 million yen, 8–10 million yen, 10–15 million yen, 15–20 million yen, ≥20 million yen, or unknown), maternal educational attainment (high school, junior college, university/graduate school, or unknown), employment status, alcohol consumption, smoking status, history of dieting before pregnancy, and engagement in ≥15 minutes of physical activity per week.

Furthermore, the questionnaire administered at 26–40 weeks included an item assessing the participants’ perceptions of GWG: ‘How important is it to avoid excessive weight gain during pregnancy?’

### Dietary survey

The sFFQ consisted of 165 food items with nine frequency categories and assessed the habitual intake of the listed foods over the last 2 months. Respondents selected one of nine frequency options: <1 time/month, 1–3 times/month, 1–2 times/week, 3–4 times/week, 5–6 times/week, once/day, 2–3 times/day, 4–6 times/day, or ≥7 times/day. For portion size, respondents selected one of three options relative to the standard portion: less than half, equal to, or ≥1.5 times the standard amount.

Food Composition in Japan (2020 edition): A weighted food composition table was created for each food item. Subsequently, the participants’ responses were used to calculate daily energy and nutrient intake, as well as intake by food groups. The validity of the sFFQ used in this study was confirmed by a 3-day dietary record survey conducted among 188 pregnant Japanese women, demonstrating the accuracy of the estimated energy and nutrient intakes.^(23)^

### Compliance score for the ‘Japanese food guide spinning top for pregnancy and lactation (JFGST-P/L)’

Dietary intake was assessed using a scoring system that measures adherence to the JFGST-P/L guidelines, which incorporate additional recommended amounts for pregnant and lactating women.^(19)^ The JFGST-P/L index indicated that these additional recommended amounts were integrated into the JFGST standard.^(13)^ Unlike systems that classify foods by nutrient or food type, the JFGST classifies foods by dish type and divides intake into five groups (grain dishes, main dishes [mainly fish/meat], side dishes [mainly vegetables], dairy products, and fruits). Intake was expressed in servings (SV). This guide also emphasizes limiting snack and beverage intake and recommends consuming no >200 kcal/day from these sources. The adherence scores were calculated based on the following seven categories: grain dishes, side dishes, main dishes, dairy products, fruits, energy from sweetness and beverages, and total energy intake. First, the SV values were calculated from each participant’s food and nutrient intake data. Furthermore, the energy intake from sweet vegetables and beverages, and total energy intake (kcal) were calculated. The definition of one SV differs by food group: one SV in grain dishes corresponds to approximately 40 g of grain-derived carbohydrates; one SV in side dishes corresponds to approximately 70 g of vegetables, mushrooms, root vegetables, legumes (excluding soybeans), and seaweed; one SV in main dishes corresponds to approximately 6 g of protein from fish, meat, eggs, or soy products; one serving of dairy products provides approximately 100 mg of calcium from milk or dairy products; and one serving of fruits is defined as 100 g of fruit. The maximum adherence score is 70 points, with up to 10 points allocated to each of the seven categories. If the intake was below or above the recommended range, the score was calculated proportionally on a scale of 0–10 points. Specifically, if intake was below the lower limit of the recommended range, the score was calculated as follows: score = 10 × (intake/lower limit of the recommended range). Conversely, if intake exceeded the upper limit, the score was calculated as: Score = 10 − 10 × [(intake − upper limit)/upper limit of recommended range]. Nutrients such as iron, calcium, and folic acid are frequently reported to be insufficient during pregnancy,^(24)^ and the recommended amounts in the food guide for pregnancy and lactation have been modified. Specifically, the upper limit for side dishes was removed, and a deduction system was applied to dairy products when calcium intake exceeded the tolerable upper intake level (for details, see Supplementary Table 1). As previously reported, the Spearman’s rank correlation coefficient between adherence scores calculated from dietary records and those from the sFFQ was 0.439 in the second and third trimesters, indicating the moderate validity of this method.^(19)^ The recommended daily intake of JFGST-P/L was determined based on age and physical activity levels. In women, physical activity levels were classified into two categories: low (I) and moderate or high (II). All the participants were classified as having moderate or high reference levels (II). This assumption was made because the data were limited and records only indicated whether participants exercised for ≥15 min per week. Furthermore, people engaged mainly in sedentary occupations are classified as having a moderate or high (II) physical activity level, as their routines include work-related movements, standing tasks, customer service, commuting, shopping, and housework.

### Determination of GWG

The total GWG of the participants was calculated by subtracting the pre-pregnancy weight from the weight at delivery. Pre-pregnancy weight was self-reported on the questionnaire. Pre-pregnancy weights and weights measured at the first prenatal check-up were not included in the study dataset. To classify GWG, we used pre-revised Japanese guidelines that were in use until 2020, the period in which the study data were collected.^(25)^ These guidelines recommend a GWG of 9–12 kg for women with a BMI <18.5, 7–12 kg for those with a BMI of 18.5–25, and approximately 5 kg for those with a BMI ≥25. Although the Japan Society of Obstetrics and Gynecology revised the recommended GWG ranges in 2021,^(26)^ earlier guidelines have long been used in epidemiological studies and are considered appropriate for ensuring comparability with existing datasets and previous research findings. Therefore, the pre-revision guidelines were adopted in this study to classify GWG.^(25)^ Body mass index (BMI) was calculated as the pre-pregnancy weight (kg) divided by height squared (m^2^). Based on the guidelines, optimal GWG was defined as 9–12 kg for women with a pre-pregnancy BMI <18.5, 7–12 kg for those with a BMI ≥18.5 and <25.0, and<5 kg for those with a BMI ≥25. Participants were categorized as excessive (above the recommended range), adequate (within the recommended range), or inadequate (below the recommended range).

### Statistical analysis

Continuous variables are expressed as mean ± standard deviation and categorical variables as percentages. As there were no clinically validated cut-off values for risk assessment based on JFGST-P/L compliance, the compliance scores were classified into quartiles (Q1–Q4) based on their distribution. Differences between the groups were evaluated using the chi-square test, and Fisher’s exact test was used when the expected frequency in any cell was less than 5. For the trend evaluation of SV counts or energy intake among the compliance score quartile groups, the Jonckheere-Terpstra test was used. With GWG categories (inadequate, adequate, and excessive) as the dependent variable, compliance scores based on the JFGST-P/L were classified into quartiles (Q1–Q4), and multinomial logistic regression analysis was performed. The quartiles were included as categorical variables in the model, with Q4 as the reference category. Using adequate GWG as the baseline, odds ratios (ORs) and 95% confidence intervals (95% CI) were calculated for inadequate and excessive GWG. To assess the linear trends, quartiles were entered into the model as continuous variables from 1 to 4, and the trend P value was calculated. Furthermore, since Q1 in the compliance score distribution was skewed toward lower values compared to the other quartiles, a binary classification analysis was conducted with Q1 as the ‘low compliance group’ and Q2–Q4 as the ‘comparison group.’ Model 1 was not adjusted for. In Model 2, adjustments were made for maternal age (years), maternal education level (high school graduate, junior college graduate, university/graduate school graduate, or unknown), primiparity, and exercise habits (at least 15 minutes per week).^(15,18,27)^ These covariates were selected a priori based on previous studies as the principal confounders in the association between diet quality and GWG. As an additional sensitivity analysis, fetal sex and gestational age were further adjusted. Multicollinearity was assessed using variance inflation factor (VIF). Using a conservative threshold of VIF <4, none of the variables exceeded this value, indicating that multicollinearity was not an issue in this analysis. Statistical analyses were conducted using SPSS for Windows version 29.0 (IBM Japan, Ltd., Tokyo), with statistical significance set at *p* < 0.05 (two-sided).

## Results

### Participants and characteristics

Among the 1,247 participants, 283 (22.7%) were classified as having inadequate GWG, 642 (51.5%) as having adequate GWG, and 322 (25.8%) as having excessive GWG. The proportion of inadequate GWG was the highest in Q1 (27.2%). The pre-pregnancy BMI categories were as follows: underweight, 254 (20.4%); normal weight, 933 (74.8%); and obese, 60 (4.8%). In addition, 69.4% of participants reported a history of dieting before pregnancy (Table 1). The baseline characteristics of the study participants are presented in Table 2.

**Table 1.** Items related to gestational weight gain by quartiles of JFGST-P/L adherence Table 1 long description.

	All	Q1 (lowest)	Q2	Q3	Q4
	*n* = 1,247	*n* = 290 (23.3)	*n* = 291 (23.3)	*n* = 328 (26.3)	*n* = 338 (27.1)
Score		7∼37	38∼43	44∼49	50∼65
Mean score ± SD	43.7 ± 8.9	31.6 ± 5.0	40.6 ± 1.7	46.6 ± 1.7	54.2 ± 3.4
GWG classification, *n* (%)					
Inadequate	283 (22.7)	79 (27.2)	60 (20.6)	65 (19.8)	79 (23.4)
Adequate	642 (51.5)	140 (48.3)	150 (51.5)	177 (54.0)	175 (51.8)
Excessive	322 (25.8)	71 (24.5)	81 (27.8)	86 (26.2)	84 (24.9)
Pre-pregnancy BMI classification, *n* (%)					
Underweight (<18.5 kg/m^2^)	254 (20.4)	47 (16.2)	71 (24.4)	67 (20.4)	69 (20.4)
Normal (18.5–24.9 kg/m^2^)	933 (74.8)	223 (76.9)	211 (72.5)	247 (75.3)	252 (74.6)
Overweight (≥25.0 kg/m^2^)	60 (4.8)	20 (6.9)	9 (3.1)	14 (4.3)	17 (5.0)
Pre-pregnancy dieting experience, *n* (%)					
YES	865 (69.4)	209 (72.1)	205 (70.4)	221 (67.4)	230 (68.0)
NO	374 (30.0)	78 (26.9)	85 (29.2)	104 (31.7)	107 (31.7)
Missing	8 (0.6)	3 (1.0)	1 (0.3)	3 (0.9)	1 (0.3)
Importance of avoiding excessive GWG, *n* (%)					
Strongly agree	479 (38.4)	113 (39.0)	113 (38.8)	127 (38.7)	126 (37.3)
Somewhat agree	676 (54.2)	154 (53.1)	157 (54.0)	181 (55.2)	184 (54.4)
Neither agree nor disagree	69 (5.5)	15 (5.2)	17 (5.8)	14 (4.3)	23 (6.8)
Somewhat disagree	11 (0.9)	4 (1.4)	2 (0.7)	2 (0.6)	3 (0.9)
Strongly disagree	2 (0.2)	1 (0.3)	1 (0.3)	0 (0.0)	0 (0.0)
Missing	10 (0.8)	3 (1.0)	1 (0.3)	4 (1.2)	2 (0.6)
Exercise ≥15 minutes/week, *n* (%)	310 (24.9)	70 (24.1)	72 (24.7)	83 (25.3)	85 (25.1)

Values are expressed as *n* (%) or mean ± SD.

**Table 2.** Baseline characteristics of participants by quartiles of JFGST-P/L adherence Table 2 long description.

	All	Q1 (lowest)	Q2	Q3	Q4
	*n* = 1,247	*n* = 290 (23.3)	*n* = 291 (23.3)	*n* = 328 (26.3)	*n* = 338 (27.1)
Compliance score (range)		7∼37	38∼43	43∼49	50∼65
Mean score ± SD	43.7 ± 8.9	31.6 ± 5.0	40.6 ± 1.7	46.6 ± 1.7	54.2 ± 3.4
Age (years)	35.9 ± 4.3	35.9 ± 4.4	35.6 ± 4.4	35.7 ± 4.2	36.4 ± 4.2
Pre-pregnancy weight (kg)	52.0 ± 7.4	52.5 ± 8.1	51.2 ± 7.0	52.1 ± 6.5	52.0 ± 7.8
GWG (kg)	9.8 ± 3.6	9.5 ± 3.9	10.1 ± 3.6	10.0 ± 3.4	9.7 ± 3.6
Pre-pregnancy BMI (kg/m^2^)	20.4 ± 2.6	20.7 ± 2.9	20.2 ± 2.5	20.4 ± 2.3	20.4 ± 2.7
Gestational age (weeks)	39.1 ± 1.4	39.0 ± 1.4	39.1 ± 1.3	39.2 ± 1.3	39.1 ± 1.7
Fetal sex, *n* (%)					
Male	654 (52.4)	158 (54.5)	155 (53.3)	165 (50.3)	176 (52.1)
Female	593 (47.6)	132 (45.5)	136 (46.7)	163 (49.7)	162 (47.9)
Primiparous, *n* (%)	778 (62.4)	176 (60.7)	187 (64.3)	209 (63.7)	206 (60.9)
Maternal education, *n* (%)					
High school	99 (7.9)	26 (9.0)	25 (8.6)	32 (9.8)	16 (4.7)
Junior college	345 (27.7)	86 (29.7)	81 (27.8)	81 (24.7)	97 (28.7)
University/graduate school	740 (59.3)	167 (57.6)	168 (57.7)	195 (59.5)	210 (62.1)
Missing	63 (5.1)	11 (3.8)	17 (5.8)	20 (6.1)	15 (4.4)
Employment status, *n* (%)					
Homemaker	508 (40.7)	119 (41.0)	123 (42.3)	121 (36.9)	145 (42.9)
Part-time employee	157 (12.6)	42 (14.5)	33 (11.3)	43 (13.1)	39 (11.5)
Self-employed	56 (4.5)	17 (5.9)	11 (3.8)	15 (4.6)	13 (3.8)
Full-time employee	426 (34.2)	93 (32.1)	100 (34.4)	119 (36.3)	114 (33.7)
Other	37 (3.0)	6 (2.1)	9 (3.1)	10 (3.0)	12 (3.6)
Missing	60 (4.8)	12 (4.1)	15 (5.2)	19 (5.8)	14 (4.1)
Annual household income, *n* (%), JPY					
<4 million	94 (7.5)	20 (6.9)	24 (8.2)	24 (7.3)	26 (7.7)
4 to <6 million	181 (14.5)	53 (18.3)	41 (14.1)	48 (14.6)	39 (11.5)
6 to <8 million	226 (18.1)	58 (20.0)	57 (19.6)	61 (18.6)	50 (14.8)
8 to <10 million	216 (17.3)	49 (16.9)	45 (15.5)	52 (15.9)	70 (20.7)
10 to <15 million	297 (23.8)	56 (19.3)	62 (21.3)	88 (26.8)	91 (26.9)
15 to <20 million	79 (6.3)	17 (5.9)	19 (6.5)	20 (6.1)	23 (6.8)
≥20 million	36 (2.9)	13 (4.5)	8 (2.7)	6 (1.8)	9 (2.7)
Missing	118 (9.5)	24 (8.3)	35 (12.0)	29 (8.8)	30 (8.9)
Smoking during pregnancy	9 (0.7)	7 (2.4)	1 (0.3)	1 (0.3)	0 (0.0)
Alcohol consumption during pregnancy	8 (0.6)	2 (0.7)	1 (0.3)	0 (0.0)	5 (1.5)

Values are expressed as *n* (%) or mean ± SD.

### Adherence to the JFGST-P/L

The mean adherence score on the JFGST-P/L was 43.7 points. The quartile distributions were as follows: Q1, 290 (23.3%); Q2, 291 (23.3%); Q3, 328 (26.3%); and Q4, 338 (27.1%). The adherence score was lowest in Q1 (31.6 points) and highest in Q4 (54.2 points), with a difference of more than 20 points between the two groups (Table 1).

### Dietary intake status

The mean number of servings (SV ± SD) for each food group was 3.4 ± 1.3 for grain dishes, 3.7 ± 2.1 for vegetable dishes, 4.6 ± 2.5 for main dishes, 2.8 ± 3.4 for dairy products, and 1.4 ± 1.4 for fruits. The intake of grains and vegetable dishes was well below the recommended levels. The mean total energy intake was 1,604 kcal, with the lowest intake observed in Q1 (1,260 kcal) (Table 3).

**Table 3.** Dietary intake and adherence scores according to quartiles of JFGST-P/L compliance Table 3 long description.

		Q1 (lowest)	Q2	Q3	Q4	
*n* = 1,247		*n* = 290 (23.3)	*n* = 291 (23.3)	*n* = 328 (26.3)	*n* = 338 (27.1)	*p*
Compliance score		7∼37	38∼43	43∼49	50∼65	
Mean ± SD	43.7 ± 8.9	31.6 ± 5.0	40.6 ± 1.7	46.6 ± 1.7	54.2 ± 3.4	<0.001
Servings (SVs) per day						
Grain dishes	3.4 ± 1.3	2.7 ± 1.4	3.2 ± 1.2	3.5 ± 1.2	4.1 ± 1.3	<0.001
Vegetable dishes	3.7 ±2.1	2.5 ± 1.9	3.2 ± 1.5	4.0 ± 2.0	4.9 ± 2.0	<0.001
Fish and meat dishes	4.6 ± 2.5	3.3 ± 2.7	4.5 ± 2.3	5.1 ± 2.6	5.3 ± 1.7	<0.001
Milk and dairy	2.8 ± 3.4	1.1 ± 2.1	1.9 ± 2.3	3.4 ± 3.5	4.4 ± 3.9	<0.001
Fruits	1.4 ±1.4	0.9 ±1.5	1.2 ± 1.3	1.4 ± 1.5	2.0 ± 1.2	<0.001
Snacks and alcoholic beverages (kcal/d)	215 ± 165	237 ± 208	217 ± 165	218 ± 164	192 ± 115	0.268
Total energy (kcal/d)	1,604 ± 521	1,260 ± 494	1,542 ± 485	1,699 ± 473	1,860 ± 442	<0.001
Adherence score (0–10 points per category)						
Grain dishes	5.7 ± 2.1	4.5 ± 1.8	5.4 ±1.9	5.9 ± 1.9	6.9 ±1.8	<0.001
Vegetable dishes	5.8 ± 2.6	4.0 ± 2.5	5.2 ± 2.2	6.3 ± 2.3	7.5 ± 2.1	<0.001
Fish and meat dishes	8.1 ± 2.5	6.3 ± 3.0	8.2 ± 2.2	8.5 ± 2.2	9.1 ± 1.6	<0.001
Milk and dairy	6.3 ± 4.0	3.0 ± 3.5	5.1 ± 3.8	7.5 ± 3.3	9.0 ± 2.1	<0.001
Fruits	3.8 ± 3.0	2.1 ± 2.3	3.0 ± 2.5	4.0 ± 2.9	5.7 ± 3.0	<0.001
Snacks and alcoholic beverages	7.7 ± 3.5	6.9 ± 4.1	7.6 ± 3.5	7.7 ± 3.4	8.5 ± 2.8	<0.001
Total energy	6.3 ± 1.8	4.8 ± 1.6	6.0 ± 1.5	6.6 ± 1.4	7.4 ±1.3	<0.001

*p*-Values are from the Jonckheere–Terpstra test.

### Comparison between the GWG groups

When comparing the three GWG groups, 79 women (27.9%) in the inadequate GWG group were classified into Q1. The GWG Inadequate group tended to have a lower JFGST-P/L adherence and a higher proportion of women who were underweight or obese before pregnancy (Table 4). Approximately 68% of all three groups had a history of dieting before pregnancy, and over 90% of all groups responded that avoiding excessive weight gain during pregnancy was necessary. Detailed information about the groups is provided in Supplementary Table 2.

**Table 4.** Comparison of maternal characteristics between GWG groups Table 4 long description.

	Inadequate	Adequate	Excessive	*p*
*n* = 1,247	283 (22.7)	642 (51.5)	322 (25.8)	
Age (years)	36.1 ± 4.3	35.9 ± 4.3	35.8 ± 8.7	0.539
Pre-pregnancy weight (kg)	53.1 ± 10.0	51.1 ± 5.8	52.7 ± 7.2	0.013
GWG (kg)	5.2 ± 2.5	9.7 ± 1.3	14.2 ± 2.1	<0.001
Pre-pregnancy BMI (kg/m^2^)	21.0 ± 3.7	20.1 ± 1.9	20.5 ± 2.5	0.258
Gestational age (weeks)	38.6 ± 1.6	39.2 ± 1.2	39.4 ± 1.5	<0.001
Primiparous, *n* (%)	163 (57.6)	407 (63.4)	208 (64.6)	0.156
Fetal sex, *n* (%)				
Male	149 (52.7)	340 (53.0)	165 (51.2)	0.878
Female	134 (47.3)	302 (47.0)	157 (48.8)	
Pre-pregnancy BMI classification, *n* (%)				
Underweight (<18.5 kg/m^2^)	81 (28.6)	110 (17.1)	63 (19.6)	
Normal weight (18.5–24.9 kg/m^2^)	170 (60.1)	524 (81.6)	239 (74.2)	<0.001
Overweight (≥25.0 kg/m^2^)	32 (11.3)	8 (1.2)	20 (6.2)	
Adherence to JFGST-P/L, *n* (%)				
Q1	79 (27.9)	140 (21.8)	71 (22.0)	
Q2	60 (21.2)	150 (23.4)	81 (25.2)	0.397
Q3	65 (23.0)	177 (27.6)	86 (26.7)	
Q4	79 (27.9)	175 (27.3)	84 (26.1)	
Servings (SVs) per day				
Grain dishes	3.3 ± 1.3	3.4 ± 1.4	3.4 ± 1.3	0.339
Vegetable dishes	3.8 ± 2.2	3.7 ± 2.0	3.7 ± 2.0	0.687
Fish and meat dishes	4.6 ± 2.5	4.6 ± 2.5	4.6 ± 2.2	0.651
Milk and dairy	2.8 ± 3.6	2.7 ± 3.1	2.9 ± 3.7	0.463
Fruits	1.4 ± 1.5	1.4 ± 1.2	1.5 ± 1.6	0.659
Snacks & alcoholic beverages (kcal/d)	213 ± 189	212 ± 153	224 ± 167	0.349
Total energy (kcal/d)	1,579 ± 525	1,599 ± 514	1,634 ± 532	0.349
Adherence score				
Total score	43.1 ± 9.7	44.0 ± 8.6	43.7 ± 8.7	0.526
Grain dishes	5.6 ± 2.1	5.7 ± 2.1	5.8 ± 2.0	0.243
Vegetable dishes	5.9 ± 2.6	5.8 ± 2.6	5.8 ± 2.7	0.694
Fish and meat dishes	7.9 ± 2.6	8.0 ± 2.5	8.3 ± 2.4	0.136
Milk and dairy	6.1 ± 4.0	6.5 ± 3.9	6.2 ± 4.0	0.261
Fruits	3.6 ± 2.9	3.9 ± 3.1	3.8 ± 3.0	0.720
Snacks and alcoholic beverages	7.8 ± 3.5	7.8 ± 3.4	7.5 ± 3.7	0.623
Total energy	6.1 ± 1.8	6.3 ± 1.8	6.4 ± 1.7	0.195

Values are presented as *n* (%) or mean ± SD unless otherwise indicated.

*p* values were calculated using the Kruskal–Wallis test for continuous variables and the chi-square test or Fisher’s exact test for categorical variables.

### Association between JFGST-P/L adherence and GWG

The association between JFGST-P/L adherence and GWG was evaluated using a multinomial logistic regression analysis (Table 5). In the dichotomized analysis (Q1 vs. Q2–Q4), women with low adherence had approximately 38% higher odds of having inadequate GWG (unadjusted model 1: OR = 1.389, 95% CI = 1.008–1.913; adjusted model 2: OR = 1.725, 95% CI = 0.823–3.613). However, in the main analysis (quartile analysis), no significant trend was observed, and the trend test in the adjusted model did not show a statistically significant difference (Inadequate: P for trend = 0.269, Excessive: P for trend = 0.711). There was no clear linearity in the changes in ORs across quartiles, suggesting that the relationship between the adherence score and GWG may not be a simple linear relationship. Moreover, no significant association was found in the subgroup analysis of women who were underweight before pregnancy (Supplementary Table 3). Similar results were obtained in sensitivity analyses (Supplementary Table 4).

**Table 5.** Association between adherence to JFGST-P/L and GWG Table 5 long description.

	Inadequate vs. Adequate GWG				Excessive vs. Adequate GWG			
	Unadjusted		Adjusted^*^		Unadjusted		Adjusted^*^	
	OR (95% CI)	*p*	OR (95% CI)	*p*	OR (95% CI)	*p*	OR (95% CI)	*p*
Q1	1.250 (0.852–1.833)	0.253	1.238 (0.842–1.821)	0.277	1.057 (0.718–1.555)	0.780	1.032 (0.700–1.522)	0.872
Q2	0.886 (0.594–1.322)	0.554	0.872 (0.583–1.306)	0.507	1.125 (0.773–1.637)	0.538	1.123 (0.770–1.639)	0.546
Q3	0.813 (0.551–1.200)	0.294	0.803 (0.542–1.188)	0.272	1.012 (0.702–1.460)	0.948	0.997 (0.690–1.441)	0.987
Q4	1		1		1		1	

	Unadjusted		Adjusted^*^		Unadjusted		Adjusted^*^	
	OR (95% CI)	*p*	OR (95% CI)	*p*	OR (95% CI)	*p*	OR (95% CI)	*p*
Q1	1.389 (1.008–1.913)	0.045	1.725 (0.823–3.613)	0.149	1.014 (0.734–1.401)	0.931	0.607 (0.234–1.570)	0.303
Q2∼Q4	1		1		1		1	

* Multivariable analyses were adjusted for maternal age, maternal educational attainment, primiparity, and regular exercise (≥15 min/week).

CI, confidence interval; OR, odds ratio.

## Discussion

Appropriate GWG during pregnancy affects birth outcomes. Previous studies have reported that inadequate GWG is associated with LBW and Small-for-gestational-age (SGA) infants, whereas excessive GWG increases the risk of macrosomia and cesarean section.^(4–7)^ Proper nutritional management throughout pregnancy is important to ensure an appropriate GWG. Achieving adequate GWG requires a well-balanced diet. In this study, total GWG was classified as inadequate, adequate, or excessive, and using the appropriate GWG as the reference category, its association with adherence to the JFGST-P/L in the second and third trimesters was examined. As a result, although no clear dose–response relationship was observed between the JFGST-P/L quartiles and GWG, in analyses with inadequate GWG as the outcome, there was a trend toward increased risk in the group with the lowest JFGST-P/L (Q1), whereas no association was observed with excessive GWG.

In Japan, the GWG guidelines were revised in 2021 to increase the recommended weight gain by approximately 3 kg.^(26)^ Concerns have been raised that the previous guideline values may have set the lower limits too low for women who were underweight or normal weight before pregnancy, and falling below these limits has been reported to increase the risk of SGA and preterm birth.^(3,28,29)^ Moreover, a GWG <8 kg increases the risk of LBW and SGA.^(30)^ Pregnant Japanese women tend to have a lower GWG than pregnant women in other countries.^(10)^ In the present study, approximately 20% of the participants failed to meet the lower limit of the former guidelines. Therefore, ‘insufficient GWG’ is a critical concern.

Regarding pre-pregnancy BMI, women classified as underweight were more likely to have inadequate GWG. In other countries, pregnant women who were underweight before pregnancy reportedly gained more weight during gestation than those with normal weight or obesity.^(31–33)^ These differing trends may reflect sociocultural factors specific to Japan, such as a strong preference for thinness and the intentional restriction of weight gain during pregnancy. Indeed, some Japanese women reported dieting during pregnancy and perceived weight gain below the recommended level as ideal.^(34,35)^ Furthermore, low nutrient intake has been demonstrated to persist from before pregnancy to gestation.^(24)^ Considering these behavioural characteristics, the inadequate GWG observed among underweight women in this study may have resulted from the combined effects of reduced dietary intake and intentional weight gain avoidance. Although no statistically significant differences were observed between the inadequate and adequate GWG groups in terms of pre-pregnancy dieting habits or perceptions of the need to avoid excessive weight gain, a higher proportion of women with inadequate GWG were categorized in Q1.

Regarding awareness of the need for pre-pregnancy dieting habits and avoidance of excessive weight gain during pregnancy, no statistically significant differences were observed among the three groups (inadequate, adequate, and excessive); however, the proportion belonging to Q1 was higher in the group with inadequate GWG. In multinomial logistic regression analysis using quartile-based analysis, no overall dose-response relationship or statistical significance was detected. In the analysis with inadequate GWG as the outcome, no increased risk was observed in the moderate-to-high dietary quality groups (Q2 and Q3), whereas in Q1, the ORs for inadequate GWG tended to be higher at 1.250 (unadjusted) and 1.238 (adjusted). Thus, although no clear association between JFGST-P/L and inadequate GWG was identified, an increased risk was suggested only for Q1. In contrast, in the analysis with excessive GWG as the outcome, no clear association, including any trend toward increased ORs, was observed in the quartile analysis. These results suggest that a lower dietary quality may be more strongly associated with inadequate GWG than with excessive GWG. Indeed, in Q1, the intake fell below the recommended levels for all food categories except snacks and beverages, suggesting a possible dependence on low-nutrient-density foods or simplified dietary patterns. In this study, the distribution of adherence scores demonstrated that insufficient intake was concentrated in Q1, whereas the intake in Q2–Q4 differed only moderately. Therefore, it was difficult to detect a linear trend across quartiles, and we believe that the association became clearer using a dichotomized model contrasting Q1, which was characterized by extremely low adherence, with the other groups.

The JFGST-P/L adherence score used in this study evaluates not only the balance among grain dishes, vegetable dishes, main dishes, dairy products, and fruits but also the appropriateness of total energy intake. This reflects the original intention of the JFGST-P/L to promote dietary patterns aligned with adequate energy intake, thus supporting the validity of the adherence score as a comprehensive indicator of dietary quality during pregnancy. Studies among young Japanese women have reported that higher adherence to the JFGST is associated with greater intakes of carbohydrates, dietary fibre, potassium, and other nutrients, including lower intakes of total fat and saturated fatty acids.^(36)^ Similar dietary behaviours during pregnancy may contribute to achieving an optimal GWG. Domestic studies examining diet patterns and GWG have used the NRF9.3 index;^(18)^ however, NRF9.3 does not capture nutrients that are abundant in staple foods, and may therefore overlook insufficient energy intake despite seemingly good diet quality. In contrast, studies in the United States have demonstrated that high adherence to the DASH diet is associated with a reduced risk of inadequate GWG,^(15)^ and its emphasis on grains, vegetables, fruits, and dairy is consistent with the direction of JFGST-P/L.

Diet during pregnancy is intricately associated with infant birth weight. Diets high in bread, sweet drinks, and soft drinks and low in fish and vegetables have been associated with LBW,^(37)^ whereas excessive intake of vegetable oils and sucrose has been linked to birth weight relative to gestational age.^(38)^ Birth weight also reflects the influence of GWG-mediated maternal energy intake.^(39)^ In this study population, the average servings of grain dishes, vegetable dishes, and fruits were far below the recommended levels, which may have contributed to the reduced total energy intake. Young Japanese women (18–29 years old) tend to have a low intake of staple foods and fruits,^(40)^ and the current findings may reflect this broader dietary pattern. Under such circumstances, since the JFGST-P/L specifies the necessary intake of food groups, it visualizes the insufficient intake of food groups prone to energy deficiency, serving as a useful intervention tool to support appropriate GWG during pregnancy. Based on these findings, the observed inadequate GWG in this study appears to be more strongly linked to quantitative deficiencies, especially in staple foods (i.e., insufficient energy intake), besides qualitative imbalances across food groups. Meanwhile, because the JFGST-P/L can assess intake status by food group, it may prove valuable as an index for identifying dietary patterns or deficiencies underlying energy shortages. This study’s findings suggest that, in nutritional guidance for pregnant women, early monitoring of adherence to the JFGST-P/L and ongoing support for dietary improvements based on guidelines throughout pregnancy are important for achieving appropriate GWG.

### Strengths and limitations

This study has several strengths. First, the use of a validated sFFQ administered to pregnant women, including those during late pregnancy, is a major strength in ensuring the accuracy of dietary intake assessments. In addition, the large sample size of 1,247 participants contributed to strong statistical power and enhanced the stability of the results.

Another strength of this study was the evaluation of dietary patterns and intake during pregnancy using the JFGST-P/L, which classifies foods based on dish categories. The JFGST-P/L is structured to help pregnant women intuitively understand ‘which food groups should be increased (or decreased), and to what extent,’ making it highly suitable for use in nutrition counseling settings. The use of a practical and widely applicable assessment tool is a notable advantage of this study. Furthermore, this study provides valuable evidence to support weight management during pregnancy by examining the association between adherence to the JFGST-P/L and GWG, specifically among women in mid-to-late pregnancy.

This study also has several limitations.

First, the data were collected from a single hospital in Tokyo, which may have limited the representativeness of the findings and restricted their generalizability.

Second, the pre-pregnancy body weight was self-reported. However, a previous study of Japanese adults (4,253 men and 1,148 women) reported a very high correlation (*r* = 0.959) between self-reported and measured body weights.^(41)^ Furthermore, self-reported pre-pregnancy body weight has been widely used in epidemiological studies, including cohort studies in Japan,^(29,42)^ and is generally considered an acceptable indicator when pre-pregnancy body weight cannot be measured. Therefore, the reliability of self-reported values was considered high. Nonetheless, the GWG is influenced by several factors, including nausea and vomiting. Although this study used an sFFQ with established external validity,^(23)^ it is possible that we were unable to fully adjust for changes in dietary intake or preferences among women experiencing nausea during pregnancy.

Third, there were limitations in the assessment of physical activity. The study only evaluated whether participants engaged in at least 15 min of physical activity per week; intensity, duration, and total energy expenditure were not assessed. Such differences may influence GWG.

Fourth, the participants tended to be older and have higher household incomes. A higher socioeconomic status has been associated with better diet quality,^(43)^ and we cannot exclude the possibility that this influenced the GWG in our study population.

Additionally, when using the JFGST for dietary assessment, the individual intake of seasonings and oils is not considered, which may lead to an overestimation of sodium intake. However, previous studies have demonstrated that higher adherence to the JFGST is associated with lower sodium excretion and lower sodium-to-potassium (Na/K) ratio,^(44)^ suggesting that this index is useful as a dietary assessment tool. Although the JFGST-P/L is advantageous for evaluating dietary patterns and amounts, it does not directly assess nutrients. Therefore, combining it with nutrient-based indices such as NRF9.3 may be desirable.

### Conclusion

Despite several limitations, this study examined the association between adherence to the JFGST-P/L and GWG among women in the mid- to-late stages of pregnancy and suggested a potential relationship. Although a clear dose-response relationship was not observed across the quartiles, the group with the lowest adherence to the JFGST-P/L tended to have an increased risk of inadequate GWG, indicating the importance of the early detection of extreme intake deficiencies. Furthermore, JFGST-P/L is useful as an indicator for assessing dietary patterns during pregnancy and the intake status of different food groups and may be utilized in nutritional counseling to support appropriate GWG. Further clarification of the applicability of the JFGST-P/L for dietary guidance during pregnancy is required through studies conducted at multiple institutions and by combining nutrient-based indicators.

## Supporting information

10.1017/jns.2026.10134.sm001 Doi et al. supplementary material Doi et al. supplementary material

## Data Availability

The data supporting the findings of this study are available upon request from the corresponding author. The data were not publicly available because of ethical restrictions.

## Supplementary material

The supplementary material for this article can be found at https://doi.org/10.1017/jns.2026.10134.

## Table 1. Long description

The table presents data on gestational weight gain (GWG) classification, pre-pregnancy BMI classification, pre-pregnancy dieting experience, importance of avoiding excessive GWG, and exercise habits among 1,247 participants. The table is divided into four quartiles (Q1 to Q4) based on JFGST-P/L adherence scores. Column headers include Score, GWG classification, Pre-pregnancy BMI classification, Pre-pregnancy dieting experience, Importance of avoiding excessive GWG, and Exercise >=15 minutes/week. Row labels include categories such as Inadequate, Adequate, Excessive for GWG classification; Underweight, Normal, Overweight for pre-pregnancy BMI; YES, NO, Missing for pre-pregnancy dieting experience; Strongly agree, Somewhat agree, Neither agree nor disagree, Somewhat disagree, Strongly disagree, Missing for importance of avoiding excessive GWG; and percentages for exercise habits. Each row provides the count and percentage of participants in each category across the quartiles. Notable trends include the highest proportion of inadequate GWG in Q1 and the majority of participants being of normal pre-pregnancy BMI across all quartiles.

Navigate back to Table 1..

## Table 2. Long description

The table presents baseline characteristics of participants categorized by quartiles of JFGST-P/L adherence. It includes data for 1,247 participants divided into four quartiles (Q1 to Q4) based on their compliance scores. The table has 17 rows and 11 columns, with row labels including compliance score, mean score, age, pre-pregnancy weight, gestational weight gain (GWG), pre-pregnancy BMI, gestational age, fetal sex, primiparous status, maternal education, employment status, annual household income, smoking during pregnancy, and alcohol consumption during pregnancy. Column headers represent the quartiles (Q1 to Q4) and the overall data (All). Each row provides specific values for each category, such as mean scores, standard deviations, and percentages.

Navigate back to Table 2..

## Table 3. Long description

The table presents dietary intake and adherence scores across quartiles of compliance, divided into four quartiles (Q1 to Q4) with varying compliance scores. It includes data on servings per day for different food groups, total energy intake, and adherence scores. Row 1: Compliance score, Q1: 7-37, Q2: 38-43, Q3: 43-49, Q4: 50-65. Row 2: Mean ± SD, Q1: 43.7 ± 8.9, Q2: 31.6 ± 5.0, Q3: 40.6 ± 1.7, Q4: 54.2 ± 3.4. Row 3: Grain dishes (SVs per day), Q1: 3.4 ± 1.3, Q2: 2.7 ± 1.4, Q3: 3.2 ± 1.2, Q4: 4.1 ± 1.3. Row 4: Vegetable dishes (SVs per day), Q1: 3.7 ± 2.1, Q2: 2.5 ± 1.9, Q3: 3.2 ± 1.5, Q4: 4.9 ± 2.0. Row 5: Fish and meat dishes (SVs per day), Q1: 4.6 ± 2.5, Q2: 3.3 ± 2.7, Q3: 4.5 ± 2.3, Q4: 5.3 ± 1.7. Row 6: Milk and dairy (SVs per day), Q1: 2.8 ± 3.4, Q2: 1.1 ± 2.1, Q3: 1.9 ± 2.3, Q4: 4.4 ± 3.9. Row 7: Fruits (SVs per day), Q1: 1.4 ± 1.4, Q2: 0.9 ± 1.5, Q3: 1.2 ± 1.3, Q4: 2.0 ± 1.2. Row 8: Snacks and alcoholic beverages (kcal/d), Q1: 215 ± 165, Q2: 237 ± 208, Q3: 217 ± 165, Q4: 192 ± 115. Row 9: Total energy (kcal/d), Q1: 1,604 ± 521, Q2: 1,260 ± 494, Q3: 1,542 ± 485, Q4: 1,860 ± 442. Row 10: Grain dishes (Adherence score), Q1: 5.7 ± 2.1, Q2: 4.5 ± 1.8, Q3: 5.4 ± 1.9, Q4: 6.9 ± 1.8. Row 11: Vegetable dishes (Adherence score), Q1: 5.8 ± 2.6, Q2: 4.0 ± 2.5, Q3: 5.2 ± 2.2, Q4: 7.5 ± 2.1. Row 12: Fish and meat dishes (Adherence score), Q1: 8.1 ± 2.5, Q2: 6.3 ± 3.0, Q3: 8.2 ± 2.2, Q4: 9.1 ± 1.6. Row 13: Milk and dairy (Adherence score), Q1: 6.3 ± 4.0, Q2: 3.0 ± 3.5, Q3: 5.1 ± 3.8, Q4: 9.0 ± 2.1. Row 14: Fruits (Adherence score), Q1: 3.8 ± 3.0, Q2: 2.1 ± 2.3, Q3: 3.0 ± 2.5, Q4: 5.7 ± 3.0. Row 15: Snacks and alcoholic beverages (Adherence score), Q1: 7.7 ± 3.5, Q2: 6.9 ± 4.1, Q3: 7.6 ± 3.5, Q4: 8.5 ± 2.8. Row 16: Total energy (Adherence score), Q1: 6.3 ± 1.8, Q2: 4.8 ± 1.6, Q3: 6.0 ± 1.5, Q4: 7.4 ± 1.3.

Navigate back to Table 3..

## Table 4. Long description

The table presents data on various health and dietary metrics across three categories of gestational weight gain: Inadequate, Adequate, and Excessive. The table has 1247 entries and includes metrics such as age, pre-pregnancy weight, gestational weight gain, pre-pregnancy BMI, gestational age, primiparous status, fetal sex, pre-pregnancy BMI classification, adherence to JFGST-P/L, servings per day, total energy intake, and adherence score. Each row provides specific values for these metrics across the three categories. Notable trends include variations in pre-pregnancy weight, gestational weight gain, and pre-pregnancy BMI classification among the categories. The table also shows adherence scores and servings per day for different food groups.

Navigate back to Table 4..

## Table 5. Long description

A table comparing inadequate and excessive gestational weight gain (GWG) with adherence to guidelines. The table has two main sections: Inadequate vs. adequate GWG and Excessive vs. adequate GWG. Each section is divided into unadjusted and adjusted models with odds ratios (OR) and 95% confidence intervals (CI) for quartiles (Q1, Q2, Q3, Q4). The first section compares inadequate GWG with adequate GWG, showing OR and p-values for each quartile in both unadjusted and adjusted models. The second section compares excessive GWG with adequate GWG, also showing OR and p-values for each quartile in both unadjusted and adjusted models. The table includes specific OR values and p-values for each comparison, indicating the statistical significance of the associations.

Navigate back to Table 5..
