# Supplementary material for: Compliance with the ‘Japanese food guide spinning top’ for pregnancy and lactation and weight gain during pregnancy
Source: J Nutr Sci. 2026 Aug 3;15:e72. doi: 10.1017/jns.2026.10134 (PMC13439021; doi:10.1017/jns.2026.10134)
Supplement: Supplementary file 1 [file S2048679026101347sup001.docx]

Supplemental Table 1. Calculation method of adherence scores to the Japanese Food Guide Spinning Top for Pregnancy and Lactation(JFGST-P/L)

| Mid-pregnancy |  | Standard for minimum score of 0 points | Standard for continuous scoring of 0-10 points*^)^ | Standard for maximum score of 10 points |
| --- | --- | --- | --- | --- |
| Grain dishes (serving/d)^†)^ | SV | 0 or ≧14 | ＞0-＜5 or ＞7-＜14 | 5－7 |
| Vegetable dishes (serving/d)）^‡)^ | SV | 0 | ＞0-＜6 | ≧6 |
| Fish and meat dishes (serving/d)^§)^ | SV | 0 or ≧12 | ＞0-＜4 or ＞6-＜12 | 4-6 |
| Milk (serving/d)^\|\|)^ | SV | 0 or ≧50 | ＞0-＜2 or ≧25-＜50 | ≧2-＜25 |
| Fruits (serving/d)^¶)^ | SV | 0 or ≧6 | ＞0-＜3or ＞3-＜6 | 3 |
| Snacks and alcoholic beverages (kcal/d) | kcal | ≧400 | ＞200-＜400 | 0-200 |
| Total energy (kcal/d) | kcal | 18~29 years old: 0 or ≧4500 | ＞0-＜2250 or ＞2250-＜4500 | 2250 |
|  |  | 30~49 years old: 0 or ≧4600 | ＞0-＜2300 or ＞2300-＜4600 | 2300 |
| Late pregnancy |  |  |  |  |
| Grain dishes (serving/d)^†)^ | SV | 0 or ≧16 | ＞0-＜6 or ＞8-＜16 | 6-8 |
| Vegetable dishes (serving/d)）^‡)^ | SV | 0 | ＞0-＜6 | ≧6 |
| Fish and meat dishes (serving/d)^§)^ | SV | 0 or ≧12 | ＞0-＜4 or ＞6-＜12 | 4-6 |
| Milk (serving/d)^\|\|)^ | SV | 0 or ≧50 | ＞0-＜3 or ≧25-＜50 | ≧3-＜25 |
| Fruits (serving/d)^¶)^ | SV | 0 or ≧6 | ＞0-＜3or ＞3-＜6 | 3 |
| Snacks and alcoholic beverages (kcal/d) | kcal | ≧400 | ＞200-＜400 | 0-200 |
| Total energy (kcal/d) | kcal | 18~29 years old: 0 or ≧4900 | ＞0-＜2450 or ＞2450-＜4900 | 2450 |
|  |  | 30~49 years old: 0 or ≧5000 | ＞0-＜2500 or ＞2500-＜5000 | 2500 |

20-49 years old: “Japanese Food Guide Spinning Top for Pregnancy and Lactation” (physical activity level: moderate or Higher II)

*）If an individual consumed less than recommended amount of servings or energy, score was calculated with formula:

Score = 10 × (intake ÷ lower limit of recommended range)

If individual consumed more than recommended amount of servings or energy, score was calculated with formula:

10 − 10 × [(intake − upper limit) ÷ upper limit of recommended range]

^†^)  Includes rice, bread, and noodles. One serving contains about 40 g carbohydrates.

^‡^) Includes vegetables, mushrooms, potatoes, and seaweed. In one serving, main ingredient weighs about 70 g. 1/2 weight for 100% vegetable juices.

^§^) Includes meat, fish, eggs, and soybeans. One serving contains about 6 g protein.

^||^) Includes milk and milk products. One serving contains about 100 mg calcium.

^¶^) In one serving, main ingredient weighs about 100 g. 1/2 weight for 100% fruit juices.

Supplemental Table 2. Comparison data between the inadequate and adequate groups

|  | **Inadequate** | | **Adequate** | | **Excessive** | | p*** |
| --- | --- | --- | --- | --- | --- | --- | --- |
| n=1,247 | n=283 | | n=642 | | n=283 | |  |
| Maternal education, n (%) |  |  |  |  |  |  | 0.677 |
| High school | 25 | (8.8) | 44 | (6.9) | 30 | (9.3) |  |
| Junior college | 80 | (28.3) | 177 | (27.6) | 88 | (27.3) |  |
| University/graduate school | 160 | (56.5) | 390 | (60.7) | 190 | (59.0) |  |
| Missing | 18 | (6.4) | 31 | (4.8) | 14 | (4.3) |  |
| Pre-pregnancy dieting experience, n (%) |  |  |  |  |  |  |  |
| YES | 195 | (68.9) | 439 | (68.4) | 231 | (71.7) | 0.502 |
| NO | 88 | (31.1) | 198 | (30.8) | 88 | (27.3) |  |
| Missing | 0 | (0.0) | 5 | (0.8) | 3 | (0.9) |  |
| Importance of avoiding excessive GWG, n (%) |  |  |  |  |  |  |  |
| Strongly agree | 110 | (38.9) | 250 | (38.9) | 119 | (37.0) | 0.269 |
| Somewhat agree | 152 | (53.7) | 353 | (55.0) | 171 | (53.1) |  |
| Neither agree nor disagree | 18 | (6.4) | 27 | (4.2) | 24 | (7.5) |  |
| Somewhat disagree | 3 | (1.1) | 3 | (0.5) | 5 | (1.6) |  |
| Strongly disagree | 0 | (0.0) | 2 | (0.3) | 0 | (0.0) |  |
| Missing | 0 | (0.0) | 7 | (1.1) | 3 | (0.9) |  |
| Exercise for ≥15 minutes per week | 59 | (20.8) | 170 | (26.5) | 86 | (26.7) | 0.305 |

Values are expressed as n (%).

p values were calculated using the Kruskal–Wallis test for continuous variables and the chi-square test or Fisher's exact test for categorical variables.

Supplemental Table 3. Association Between Adherence to JFGST-P/L and GWG (Underweight )

|  | Inadequate vs. Adequate GWG | | | | Excessive vs. Adequate GWG | | | | | |
| --- | --- | --- | --- | --- | --- | --- | --- | --- | --- | --- |
|  | Unadjusted |  | Adjusted * |  | | Unadjusted |  | Adjusted * |  |  |
|  | OR（95％CI） | p | OR（95％CI） | p | | OR（95％CI） | p | OR（95％CI） | p |  |
| Q1 | 1.457  （0.634-3.348） | 0.375 | 1.524  （0.636-3.652） | 0.344 | | 0.594  （0.208-1.692） | 0.329 | 0.599  （0.205-1.752） | 0.349 |  |
| Q2 | 0.976  （0.438-2.175） | 0.953 | 0.945  （0.413-2.161） | 0.894 | | 1.432  （0.640-3.204） | 0.382 | 1.398  （0.612-3.192） | 0.427 |  |
| Q3 | 0.678  （0.306-1.500） | 0.337 | 0.737  （0.323-1.685） | 0.470 | | 0.644  （0.274-1.514） | 0.314 | 0.654  （0.271-1.579） | 0.345 |  |
| Q4 | 1 |  | 1 |  | | 1 |  | 1 |  |  |

|  | Unadjusted |  | Adjusted * |  | Unadjusted |  | Adjusted * |  |
| --- | --- | --- | --- | --- | --- | --- | --- | --- |
|  | OR（95％CI） | p | OR（95％CI） | p | OR（95％CI） | p | OR（95％CI） | p |
| Q1 | 1.676  （0.832-3.379） | 0.149 | 1.725  （0.823-3.613） |  | 0.599  （0.237-1.515） | 0.279 | 0.607  （0.234-1.570） | 0.303 |
| Q2～Q4 | 1 |  | 1 |  | 1 |  | 1 |  |

p values were calculated using the Kruskal–Wallis test for continuous variables and the chi-square test or Fisher's exact test for categorical variables.

Supplemental Table 4. Association Between Adherence to JFGST-P/L and GWG (Infant sex,　Gestational age)

|  | Inadequate vs. Adequate GWG | | | | Excessive vs. Adequate GWG | | | | | |
| --- | --- | --- | --- | --- | --- | --- | --- | --- | --- | --- |
|  | Model1 |  | Model2 |  | | Model1 |  | Model2 |  |  |
|  | OR（95％CI） | p | OR（95％CI） | p | | OR（95％CI） | p | OR（95％CI） | p |  |
| Q1 | 1.239  （0.843-1.822） | 0.276 | 1.226  （0.829-1.814） | 0.307 | | 1.034  （0.702-1.525） | 0.865 | 1.045  （0.708-1.544） | 0.823 |  |
| Q2 | 0.873  （0.583-1.306） | 0.508 | 0.889  （0.590-1.337） | 0.571 | | 1.124  （0.770-1.640） | 0.545 | 1.126  （0.770-1.654） | 0.541 |  |
| Q3 | 0.802  （0.542-1.187） | 0.271 | 0.841  （0.565-1.251） | 0.392 | | 0.996  （0.689-1.440） | 0.982 | 0.978  （0.676-1.416） | 0.907 |  |
| Q4 | 1 |  | 1 |  | | 1 |  | 1 |  |  |

|  | Model1 |  | Model2 |  | Model1 |  | Model2 |  |
| --- | --- | --- | --- | --- | --- | --- | --- | --- |
|  | OR（95％CI） | p | OR（95％CI） | p | OR（95％CI） | p | OR（95％CI） | p |
| Q1 | 1.389  （1.006-1.918） | 0.046 | 1.347  （0.973-1.867） | 0.074 | 0.999  （0.722-1.382） | 0.995 | 1.015  （0.733-1.407） | 0.927 |
| Q2～Q4 | 1 |  | 1 |  | 1 |  | 1 |  |

Model 1: Adjusted for maternal age, maternal educational attainment, primiparity, regular exercise (≥15 min/week), and infant sex.

Model 2: Adjusted for maternal age, maternal educational attainment, primiparity, regular exercise (≥15 min/week), and Gestational age.
